# Preparation and preliminary quality evaluation of aspirin/L-glutamate compound pellets

**DOI:** 10.1007/s10856-021-06594-8

**Published:** 2021-08-30

**Authors:** Mengchang Xu, Fenglin Liu, Wenhu Zhou, Binsheng He, Songwen Tan

**Affiliations:** 1 Academician Workstation, Changsha Medical University, Changsha, 410219 China; 2 Xiangya School of Pharmaceutical Sciences, Central South University, Changsha, 410013 China

## Abstract

L-glutamate is an important component of protein. It can prevent gastrointestinal damage caused by NSAIDs. We constructed two-phase enteric-coated granules of aspirin and L-glutamate compound by extrusion spheronization method and fluidized bed coating. The subliminal effective dose of L-glutamate is 100 mg/kg tested by model of gastric ulcer of rats induced by aspirin and drug administration. HPLC-UV and UV–Vis methods were adopted to determine content and cumulative release of aspirin and L-glutamate as quality analysis method indexes. The prescription and process optimization were carried out with yield, sphericity and dissolution. The two-phase compound granules have good sphericity of 0.93 ± 0.05 (aspirin pellets) and 0.94 ± 0.02 (L-glutamate pellets), content of salicylic acid (0.24 ± 0.03)%, dissolution of aspirin (2.36 ± 0.11)%. Quality evaluation and preliminary stability meet the commercial requirements. The stored environment of compound preparation should be sealed in a cool and dark place.

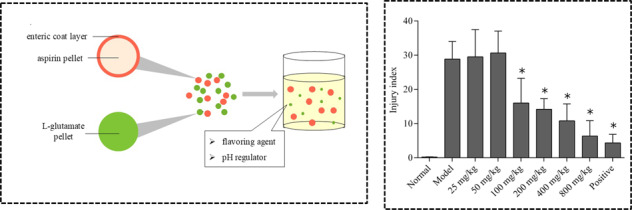

## Introduction

Non-steroidal anti-inflammatory drugs (NSAIDs) are widely available to treat fever, pain, and arthritis, which is one of the most frequently prescribed drugs in the world [1]. NSAIDs exert their effects by inhibiting the activity of cyclooxygenase (COX). Common COX has two subtypes: COX-1 and COX-2. COX-1 is necessary to maintain certain normal functions of human body and participates in the synthesis of the prostate (PGs) required for normal cell activities. COX-2 increases expression under tissue injury and inflammation, while participates in the synthesis of mediate inflammation, pain, PGs. NSAIDs exert antipyretic, analgesic, and anti-inflammatory effects by inhibition of COX-2 activity, while exert antithrombotic effect by inhibition of COX-1 activity. According to the inhibitory mechanisms for COX, NSAIDs are divided into COX-1 high-selection inhibitors (such as, aspirin, indomethacin), COX-1 low-selection inhibitors (ibuprofen, acetaminophen), COX non-selection inhibitors (naproxen, diclofenac), COX-2 selection inhibitors (celecoxib, rofecoxib), and so on [2]. However, NSAIDs can cause severe gastrointestinal adverse reactions using for a long-term. Epidemiological studies found that about 20–30% of patients appear gastric ulcers and 2% suffer severe complications such as gastric bleeding or perforation and even death. The incidence of high-risk groups can reach to 10% [3]. The incidence of dyspepsia is 1.5–2 times that of non-drugs using [4]. About 5–15% of patients with rheumatoid arthritis discontinue drugs due to gastrointestinal adverse reactions [5]. It can be seen that the gastric injury effect of NSAIDs has become a huge obstacle to the clinical use of such drugs, and it is of great clinical significance to find a safe and effective way to prevent gastric injury caused by NSAIDs.

At present, the common methods to prevent gastric injury caused by NSAIDs include the combination use of antiulcer drugs or mucosal protection drugs, or use of selection COX-2 inhibitors, dual pathway inhibitors of COX and 5-LOX [6]. The most common anti-ulcer drug to protect gastric injury caused by NSAIDs is proton pump inhibitor (PPI). PPI can effectively inhibit incidence of gastric ulcers caused by NSAIDs, while long-term use of PPI can cause fractures (hip, wrist, and spinal) and gastrointestinal microbial homeostasis [7–9]. Gastric mucosal protective drugs such as misoprostol can effectively reduce the gastric ulcer caused by NSAIDs, but the effect on dyspepsia is poor which limits its application [10]. COX-2 selection inhibitors can significantly reduce gastrointestinal adverse reactions [11]. COX and 5-COX dual pathway inhibitors have anti-inflammatory and analgesic effects almost no gastrointestinal side effects, but benzoxprofen was withdrawn from global market due to severe hepatotoxicity [12]. Now, there is still no ideal approach to gastric injury resistance caused by NSAIDs.

In recent years, researchers have shown that L-glutamic has gastrointestinal protection and functional regulation through multiple pathways including increasing mucus and bicarbonate secreation [13], increasing mucin expression, enhancing intercellular adhesion, inhibiting microbial invasion [14, 15], enhancing the gastrointestinal mucosal barrier effect, reducing intestinal oxidative stress-induced damage, and promoting the repair of gastric damage [16, 17]. In addition, L-glutamic is relatively safe for individual even pregnant women, lactating women and children [18]. Thus, L-glutamic is considered as a potential drugs which is an effective and safe way to reduce gastric mucosal damage caused by NSAIDs. In this work, we proposed effective dose of L-glutamate using aspirin as a model drug with stomach injury model of rats for gastrointestinal protection. We constructed a biphasic release compound formulation of aspirin (enteric) and L-glutamate and studied the prescription and process of compound preparation. This study provides a novel idea for protection of gastric mucosa damage caused by NSAIDs.

## Materials and methods

### Chemicals

Aspirin (>100%) was pharmaceutical grade and purchased from Shandong Xinhua Pharmaceutical Co., Ltd., China. L-glutamate (>98.5%) was pharmaceutical grade and purchased from Jizhou Huayang Chemical Co., Ltd., Salicylic acid (>99.9%) was pharmaceutical grade and purchased from China National Institute for Food and Drug Control. Sodium carboxymethyl cellulose, PVP K30, and hypromellose were AR grade and purchased from Anhui Sunward Pharmaceutical Excipients Co., Ltd., China. Hydrochloric acid, ethanol (95%), and tartaric acid were analytical grade and purchased from Sinopharm Chemical Reagent Co., Ltd., China. Glyceryl monostearate (GMS) was AR grade and purchased from Hunan Huihong Reagent Co., Ltd., China. Steria was pharmaceutical grade and purchased from Qufu Shengren Pharmaceutical Co., Ltd., China. All chemicals were used without further purification.

### Effective dosing of L-glutamate

#### Construction of stomach damage model

Twenty-four SD rats were randomly divided into six groups: blank control group, aspirin 25, 50, 100, 200, 400 mg/kg group, respectively. Aspirin was intragastric administrated for 7 days and once a day. The statistic data from designed experiments, see Table. 1. The rate of gastric ulcer was 100% when the dose of aspirin was equal or greater than 200 mg/kg. Therefore, we use 200 mg/kg aspirin to build gastric injury model of rats.

**Table 1:** Lesion index of gastric mucosal and rate of gastric ulcer of rats induced by aspirin (Mean ± SD, *n* = 4)

Dose of aspirin (mg/kg)	Lesion index	Rate of gastric ulcer (%)
0	0	0
25	0	0
50	0.75 ± 1.50	25
100	4.50 ± 3.42	75
200	25.50 ± 2.65	100
400	29.50 ± 4.36	100

#### Effective dosing of L-glutamate for gastric injury protection

**Fig. 1:**
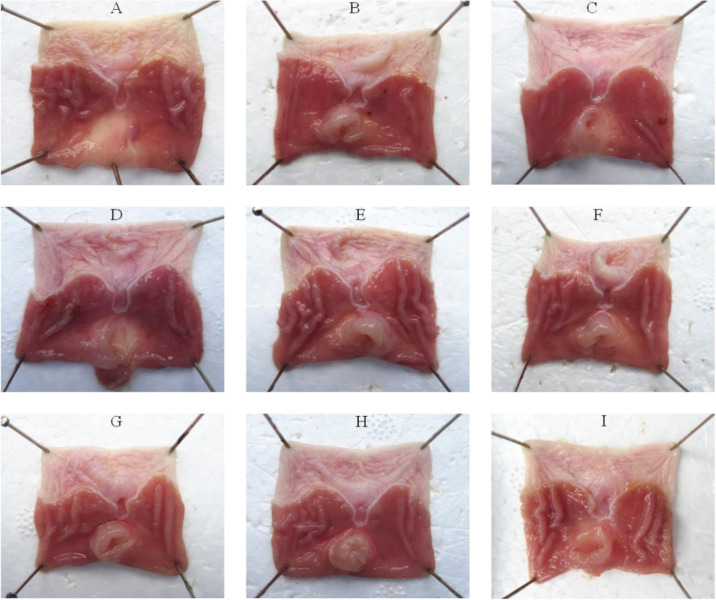
The general morphology of gastric mucosa of rats. **A** Normal group, **B** Model group, **C** L-Glutamate 25 mg/kg, **D** L-Glutamate 50 mg/kg, **E** L-Glutamate 100 mg/kg, **F** L-Glutamate 200 mg/kg, **G** L-Glutamate 400 mg/kg, **H** L-Glutamate 800 mg/kg, **I** Positive control group

**Fig. 2:**
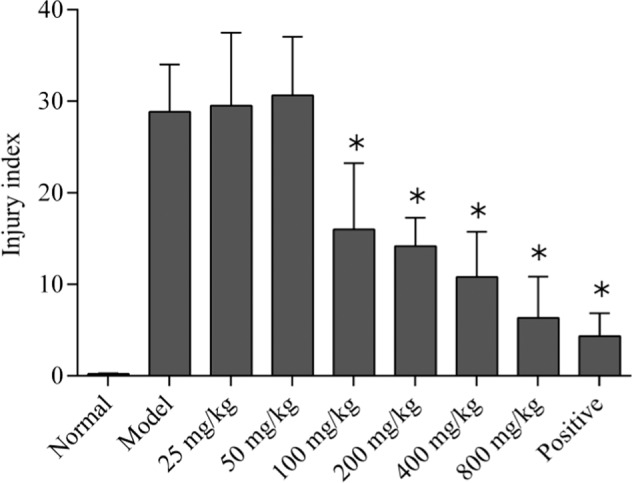
Effect of L-glutamate on gastric mucosal lesion index in rats induced by aspirin. *Significantly different from the model at *p* < 0.01

Seventy-two SD rats were randomly divided into nine groups: blank control group, model group, L-glutamate group (25, 50, 100, 200, 400, 800 mg/kg), and positive control group (ranitidine hydrochloride, 30 mg/kg) (see Table S1). The drugs were intragastric administrated for 7 days and once a day (Tables 2 and 3). The gastric scale was tested to evaluate the injury protection performance of different doses (see Fig. 1) and statistic data of inhibition rate were shown in Table S2. The results showed that the protective effect was weak when L-glutamate was 25 and 50 mg/kg (på 0.5). When the dose of L-glutamate was equal or greater than 100 mg/kg, its protective effect was significant (*p* < 0.01) with a dose-dependent (see Fig. 2). The analysis results of tissue morphology as evaluation index (see Fig. S1) kept the consistency.

**Table 2:** The composition of prescription of aspirin drug-loaded pellets

Composition	The prescription analysis	Amount (%)
Aspirin	API	44
MCC	Filler	44
L-HPC	Disintegrant	5
PVP K30	Adhesive	5
Tartaric acid	Stabilizer	2
Water	Moistening agent	–
Ethanol	Moistening agent	–

**Table 3:** The parameter of extrusion-spheronization process of aspirin drug-loaded pellets

Parameter	Value
Extrusion speed	30 rpm
Spheronization speed	1000 rpm
Spheronization time	4 min

### The dosage form of compound preparation

Low-dose aspirin (75–150 mg) is often used for preventing secondary cardiovascular disease. In this paper, specification of aspirin is 100 mg which refer to aspirin preparation, just like “Bayaspirin” (100 mg) and “Axanum” (81 mg). The subliminal effective dose of L-glutamate is 100 mg/kg which is equal 1 g for human. So the specification of L-glutamate is 1 g [19].

**Fig. 3:**
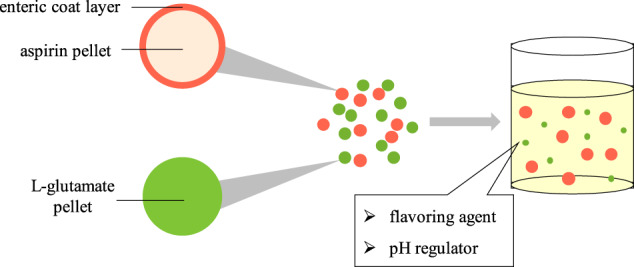
The schematic diagram of aspirin and L-glutamate compound enteric-coated granules

Aspirin enteric-coated preparations can significantly reduce the direct damage for gastric mucosa. Therefore, aspirin would be regarded as enteric preparation. There are many advantages of pellets, such as little effect by gastric emptying, drug absorption evenly, local irritation for gastrointestinal slightly, individual pellet defects having little impact on preparation, conducive to coating, flexible control of drug dose and so on. Thus, the compound preparation is prepared as pellets. According to the preliminary test results, the density of L-glutamate pellets is about 0.6 g/ml. If the preparation is made into capsules, 4–5 capsules of No. 0 capsules are need. So the drug adaptability is poor. The compound preparation was prepared as a two-phase release pellet granule, i.e., aspirin enteric pellets and L-glutamic acid common pellets (Fig. 3).

### The establishment of quality analysis methods

The content and cumulative release of aspirin and L-glutamate compound enteric granules are important evaluation indexes. Aspirin is easy hydrolyzed to irritating salicylic acid. So, the amount of salicylic acid should be controlled to ensure the quality of the preparation and medication safety.

HPLC-UV methods were used to determine the content and release of aspirin and the content of salicylic acid. A Diamonsil C18 column (250 × 4.6 mm, 5 μm) was used with the mobile phase consisting of acetonitrile-tetrahydrofuran-glacial acetic acid-water (20:5:5:70). The detection wavelength of content and release of aspirin is 276 nm. The detection wavelength is 303 nm of the content of salicylic acid. Results from chromatograms, standard curve, accuracy curve, repeatability, and recovery experiments showed that the methods were sensitive, effective, and specific used as quantitative analysis.

UV–Vis was used to determine the content and dissolution of L-glutamate. Ninhydrin was used as the color reagent and the derivative is stability. The maximum absorption wavelength is 567 nm. Results from repeatability, recovery, and stability tests showed that the method was sensitive, effective, and specific.

## Results and discussion

### Formulation and process study

#### Aspirin enteric pellets

Extrusion-spheronization method was used to prepare aspirin pellets [20]. The prescription and process optimization were carried out with yield, sphericity, and dissolution as evaluation indexes. The prescription factors include the amount of filler, disintegrant and stabilizer and the type of wetting agent. The process factors include extrusion speed, spheronization speed, spheronization time, and fluidized bed drying time [21–24].

The inquiry experiment results indicated that the optimal formulation included aspirin (44%), microcrystalline cellulose (MCC, 44%), L-HPC (5%), PVP K30 (5%), tartaric acid (2%), and the optimal process were 30 rpm of extrusion speed and 1000 rpm of spheronization rate in 4 min. Pellets were prepared by the optimal formulation and process that performed high yield of (86.77 ± 3.65)% and rapid dissolution rate of (83.30 ± 0.23)% at 30 min in pH 6.8 PBS (In details, see Tables S3 and S4 and Fig. S2).

The enteric-coated layers with Eudragit L30D-55 including plasticizer triethyl citrate (10%) and antiadherent GMS (5%) were coated on aspirin pellets by fluidized bed method [25, 26]. The detailed composition pf enteric coating, see Table 4. The enteric layer was prepared with process parameters: 5 rpm of spray rate, 0.09 MPa of atomization pressure, 40 °C of the inlet temperature, 32–35 °C of the material temperature (see Table 5). When aspirin pellets were coated by enteric-coated layer with 10% weight gain the drug release percentage was (1.77 ± 0.08)% in pH 1.0 hydrochloric acid solution for 2 h and (88.76 ± 0.76)% in pH 6.8 PBS for 45 min. The evaluation results see Table 6 and Fig. S3. It shows that the result meets the requirements that drug release in the intestinal.

**Table 4:** The composition prescription of enteric coating solution

Composition	The prescription analysis	Amount
Eudragit L30D-55	Enteric material	50
GMS	Antiadherent	0.75
TEC	Plasticizer	1.5
Tween-80	Stabilizer	0.3

**Table 5:** The coating parameters of aspirin enteric pellets

Parameter	Value
The temperature of material	32–35 °C
The temperature of air inlet	40 °C
The spray flow	5 rpm
The pressure of atomization	0.09 MPa
The frequency	1300–1450 rpm

**Table 6:** The effect of the weight gain of enteric layer on release behavior of aspirin enteric-coated pellets in 0.1 mol/l HCl solution

The weight gain (%)	Cumulative release (%)
10	1.77 ± 0.08
15	1.62 ± 0.56
20	0.98 ± 0.26

#### L-glutamate pellets

L-glutamate pellets were prepared by extrusion-spheronization method and the prescription and process optimization were carried out with yield, sphericity, and dissolution as the main evaluation indexes. The prescription factors include the amount of filler, disintegrant and flavoring agent, and the type of adhesive. The results indicated that the optimal formulation included L-glutamate (68%), MCC (18%), L-HPC (6%), hypromellose (HPMC, 2%), and stevia (6%) (see Table 7). The optimal process (see Table 8) were 800 rpm × 2 min + 500 rpm × 2 min of spheronization process and 500 rpm of inlet air speed in 3 min. Pellets were prepared by the optimal formulation and process that performed high yield of (85.80 ± 3.68)% and rapid dissolution of (98.43 ± 1.05)% at 30 min in pH 7.2 PBS.

**Table 7:** The composition of prescription of L-glutamate drug-loaded pellets

Composition	The prescription analysis	Amount (%)
L-Glutamate	API	68
MCC	Filler	18
L-HPC	Disintegrant	6
HPMC	Adhesive	2
Steviosin	Flavoring agent	6
Water	Moistening agent	–

**Table 8:** The parameter of extrusion-spheronization process of L-glutamate drug-loaded pellets

Parameter	Value
Extrusion speed	30 rpm
Spheronization speed and time	800 rpm × 2 min 500 rpm × 2 min
Wind speed and time	500 rpm × 3 min

#### Mixing process of compound preparations

Due to the mass of the pellets are small (100 g), we mixed the pellets manually. The aspirin enteric-coated pellets were placed in a sealed bag since the instability of moisture. Simulating a three-dimensional mixed form, L-glutamate pellets were added by the method of equivalent addition and mixed at the same time. Finally, the mixture pellets of compound formulation can be obtained.

### Quality evaluation and preliminary stability study

The rationality and procedure reproducibility of prescription preparation was studied to evaluate the quality of products by few factors: determination of appearance, sphericity, size distribution, bulk density, free salicylic acid, uniformity of aspirin content, and release of aspirin.

**Fig. 4:**
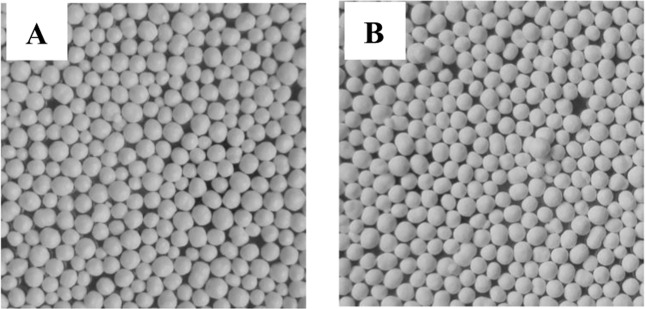
The appearance of the aspirin (**A**) and L-glutamate (**B**) drug-loaded pellets (visual inspection)

**Fig. 5:**
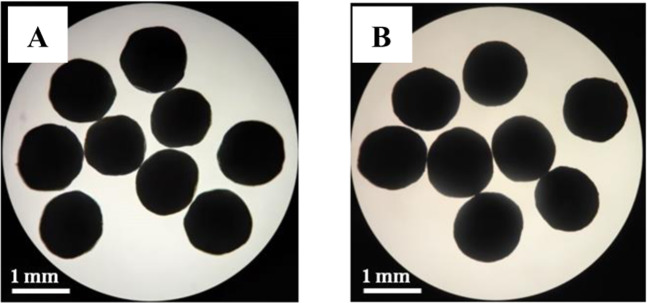
Optical micrographs of the aspirin (**A**) and L-glutamate (**B**) drug-loaded pellets

Figure 4 showed that both aspirin enteric-coated pellets and L-glutamic acid pellets are white spherical pellets with good appearance. The sphericity of pellets was described by evaluation ratio of the short diameter and long diameter: randomly take 20 pellets, place them under an optical microscope (Fig. 5), then measure the short diameters and long diameters, and calculate the ratio [27], the results are shown in Table 9. The results showed aspirin enteric-coated pellets and L-glutamate pellets were white spherical pellets and sphericity was 0.93 ± 0.05 (aspirin pellets) and 0.94 ± 0.02 (L-glutamate pellets), respectively.

**Table 9:** The sphericity of aspirin and L-glutamate drug-loaded pellets

Batch number	Aspirin pellets	L-Glutamate pellets
1	0.95 ± 0.03	0.94 ± 0.02
2	0.93 ± 0.07	0.93 ± 0.03
3	0.92 ± 0.04	0.94 ± 0.02
Mean	0.93 ± 0.05	0.94 ± 0.02

According to pharmacopoeia regulations [28], *A* = |100 − Mean|, *A* + 2.2S = 0.343 + 2.2 × 6.24 = 14.06, unless otherwise specified, *L* = 15.0, if *A* + 2.2S ≤ *L*, the content uniformity of the test product meets the requirements. Therefore, the content uniformity of aspirin in the compound preparation meets the requirements (see Table 10). The test results showed that the content of salicylic acid was (0.24 ± 0.03)%, which was <1.0% in Table 11, which meets the quality requirements. In Table S5 and Fig. S4, the dissolution of aspirin was (2.36 ± 0.11)% in pH 1.0 for 2 h and (83.29 ± 2.01)% in pH 6.8 PBS for 45 min. The dissolution of L-glutamate pellets was (98.92 ± 0.88)% in pH 7.2 PBS for 45 min in Fig. S5. The analysis results of dissolution of aspirin and L-glutamate also meet the quality requirements.

**Table 10:** Content uniformity of aspirin in compound enteric-coated granules

No	Content (%)
1	89.48
2	99.39
3	104.15
4	102.51
5	108.74
6	95.52
7	102.30
8	107.47
9	92.02
10	102.51
Mean ± SD	100.34 ± 6.24

**Table 11:** The content of salicylic acid in compound enteric-coated granules

Batch number	Salicylic acid (%)
1	0.24 ± 0.02
2	0.24 ± 0.03
3	0.26 ± 0.05

According to the general rules for the determination of content uniformity, the content uniformity of aspirin in the compound enteric-coated granules meets the requirements, but its content exceeds the limit specified in the Pharmacopoeia for aspirin enteric-coated tablets and aspirin enteric-coated capsules (93.0–107.0%), the main reason is that the doses of two drugs are quite different, thus, particle size distribution and bulk density of the two pellets are different.

**Fig. 6:**
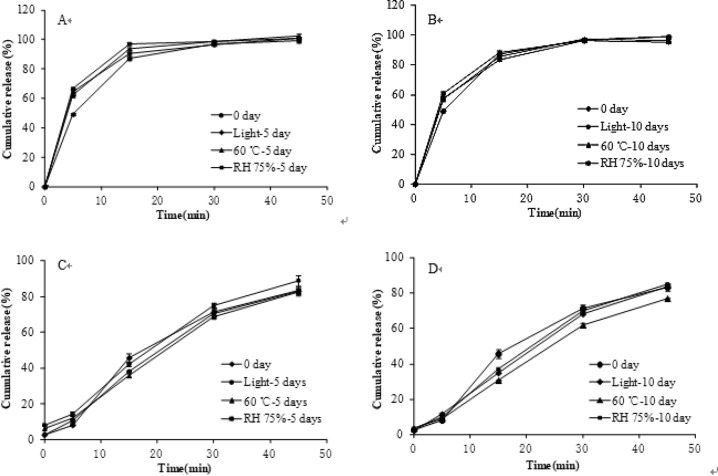
The stress testing results of cumulative release of compound enteric-coated granules (**A**, **B** release of L-glutamate; **C**, **D** release of aspirin)

The results of influencing factors tests showed that the free salicylic acid was increased to (6.54 ± 0.07)% at a high temperature (60 °C) for 10 days which exceeding the standard of 1.0% (Table 12) and the cumulative release of aspirin in pH 6.8 PBS for 45 min was <80% (Fig. 6). It means that the compound enteric-coated granules is unstable under high temperature conditions. Under the other conditions for 10 days quality meet the requirements. Therefore, the compound preparation should be sealed in a cool and dark place.

**Table 12:** The stress testing results of compound enteric-coated granules

Condition	Characters	Weight change (%)	Salicylic acid (%)	Acid resistance (%)
0 day	White spherical pellet	–	0.24 ± 0.02	2.30 ± 0.10
5 day				
60 °C	White spherical pellet	−1.37 ± 0.05	4.26 ± 0.31	3.54 ± 0.34
RH 75%	White spherical pellet	2.17 ± 0.02	0.64 ± 0.05	3.34 ± 0.17
4500 lx	White spherical pellet	−0.03 ± 0.07	0.52 ± 0.04	2.36 ± 0.24
10 day				
60 °C	White spherical pellet	−1.67 ± 0.13	6.54 ± 0.07	6.15 ± 0.34
RH 75%	White spherical pellet	2.19 ± 0.05	0.56 ± 0.05	8.04 ± 0.22
4500 lx	White spherical pellet	−0.04 ± 0.04	0.83 ± 0.04	2.91 ± 0.10

There is no apparent change in shape of compound enteric-coated granules when placed at 92.5% humidity for 5 days. But weight gain was (5.18 ± 0.05)% caused by moisture absorption. When humidity condition was changed to a relative humidity of 75%, the weight gain of 5 and 10 days were (2.17 ± 0.02)% and (2.19 ± 0.05)%, respectively. When placed at high temperature (60 °C) for 10 days, the weight of free salicylic acid increased to (6.54 ± 0.07)%. Therefore, the storage temperature should not be too high. The results of this study are slightly different from other studies of aspirin stability [29]. Thus, further research work about the stability of this compound preparation should be done.

### Particle size distribution and bulk density

The specifications of two pellets in the compound preparation are quite different. The consistency of the particle size distribution and bulk density is of great significance to the uniformity of pellets. In this article, the particle size distribution and bulk density of two pellets are quite different, so the determination of content uniformity for aspirin is quite necessary.

### Release of drugs

When tested the acid release of compound enteric-coated granules, a small amount of aspirin enteric-coated pellets is dissolved. The release rate of the two pellets meets the requirements with non-interference each other during the determination test.

## Conclusion

This study shows that L-glutamate has obvious protection effect on chronic gastric injury caused by NSAIDs like aspirin. The minimum effective dose of L-glutamate is 100 mg/kg. The two-phase compound enteric granules can be prepared by adopting the combination technique of extrusion spheronization method and fluidized bed coating. The test results of prepared pellets showed that appearance pellets is white spherical structure. And yield can reach to over 85%. The release of free salicylic acid is in line with the quality requirement. Meanwhile, the preparation process is simple and has good reproducibility. The influencing factors tests evident that the compound enteric-coated granules should be sealed in a cool and dark place. The method can provide a guide for development of compound formulations in the future.

## Supplementary information

Supplementary information
