# Supplementary material for: Preparation and preliminary quality evaluation of aspirin/L-glutamate compound pellets
Source: J Mater Sci Mater Med. 2021 Aug 30;32(9):116. doi: 10.1007/s10856-021-06594-8 (PMC8405465; doi:10.1007/s10856-021-06594-8)
Supplement: Supplementary file 1 — Supplementary information [file 10856_2021_6594_MOESM1_ESM.docx]

**Preparation and preliminary quality evaluation of aspirin/L-glutamate compound pellets**

Mengchang Xu ^a^, Fenglin Liu ^b^, Wenhu Zhou ^a, b, *^, Binsheng He ^a^, Songwen Tan ^a, b, *^

a Academician Workstation, Changsha Medical University, Changsha 410219, China

b Xiangya School of Pharmaceutical Sciences, Central South University, Changsha 410013, China

*Corresponding author: zhouwenhu@csu.edu.cn; stan0309@uni.sydney.edu.au

72 male SD rats were randomly divided into 9 groups with 8 rats in each group: Blank control group (group A), model group (group B), L-glutamic acid group (C: 25 mg/kg, D: 50 mg/kg, E: 100 mg/kg, F: 200 mg/ kg, G: 400 mg/kg, H: 800 mg/kg), positive control group (group I) (ranitidine hydrochloride, 30 mg/kg), The statistic data from designed experiments see Tab.S1. Intragastric administration once a day for 7 consecutive days with a volume of 1 mL each time. Fasting for 8 hours before gavage.

Tab.S 1 The group of rats and drug administration

| Group | | Dose (mg/kg) | | |
| --- | --- | --- | --- | --- |
|  |  | Aspirin | L-Glutamate | Ranitidine |
| Nomal | A | - | - | - |
| Model | B | 200 | - | - |
| Experimental group | C | 200 | 25 | - |
|  | D | 200 | 50 | - |
|  | E | 200 | 100 | - |
|  | F | 200 | 200 | - |
|  | G | 200 | 400 | - |
|  | H | 200 | 800 | - |
| Positive control | I | 200 | - | 30 |

Tab.S2 showed that gastric mucosa of the blank control group has no ulcers and bleeding, and the injury index is 0; the model group has a large number of bleeding in the gastric mucosa, the injury index is about 27; L-glutamic acid administration of 25 mg/kg and 50 mg/kg has a large number of bleeding, the damage index is not significantly different from the model group (p>0.5), and the protective effect is weak; when the dose of L-glutamic acid is greater than or equal to 100 mg/kg, the damage index is significantly lower than that of the model group ( p<0.01), the protective effect is strong, and the protective effect increases with increasing dose.

Tab.S 2 Protection of L-glutamate on gastric mucosal lesion of rats induced by aspirin

| Group | | Lesion index | Inhibition rate（%） |
| --- | --- | --- | --- |
| Nomal | A | 0 | - |
| Model | B | 27.17 ± 2.64 | - |
| Experimental group | C | 27.00 ± 6.32 | 0.6 |
|  | D | 28.17 ± 4.71 | - 3.7 |
|  | E | 15.17 ± 4.58 | 44.2 |
|  | F | 11.67 ± 3.20 | 57.0 |
|  | G | 6.83 ± 4.36 | 74.9 |
|  | H | 4.50 ± 3.45 | 83.4 |
| Positive control | I | 3.00 ± 2.28 | 89.0 |

Fig.S1 showed blank control group has normal gastric mucosal tissue morphology, complete epithelial structure, well-defined hierarchy, complete gastric pits and gastric gland structure, and damage grade is 0; the model group and the L-glutamate 25, 50 mg/kg group have a large number of epithelial cell necrosis and destroyed gland, a large number of vacuolar cells are seen, and the damage grade is Ⅱ-Ⅲ. In the L-glutamic acid of 100 mg/kg and 200 mg/kg groups, a small amount of epithelial cells fell off, the gastric pits and gastric glands are intact, and some cells are vacuolated. The damage grade is Ⅰ-Ⅱ. In the L-glutamic acid of 400 mg/kg and 800 mg/kg groups and the positive control group, the mucosal epithelial cells are basically intact, the gastric pits and gastric glands are intact. The damage grade was 0-I. This confirmed at the tissue level that L-glutamic acid doses greater than or equal to 100 mg/kg have a significant protective effect on aspirin-induced gastric injury in rats, and it is dose dependent.


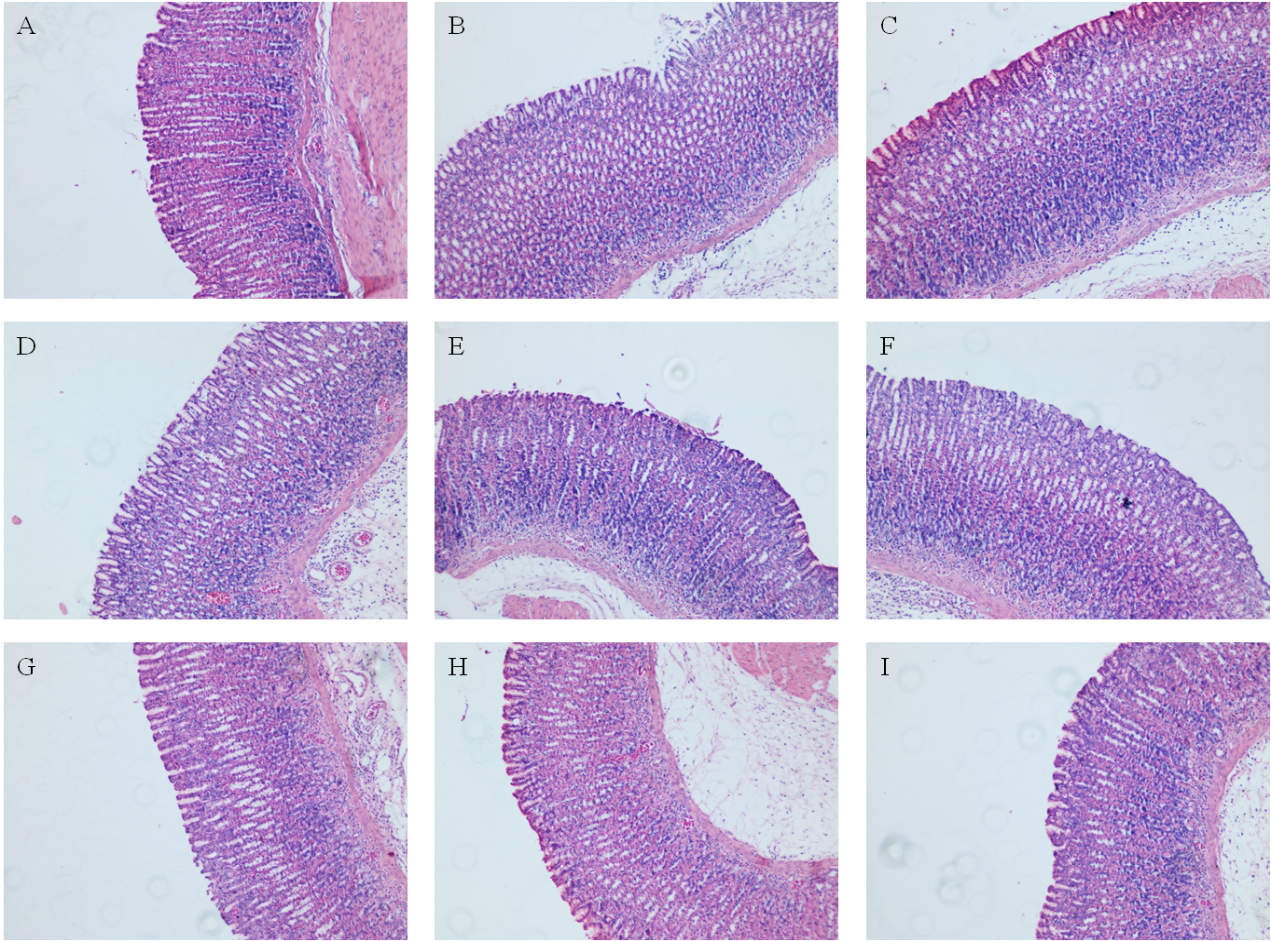


Fig.S 1 Histopathologic of gastric mucosal tissue in rats (HE staining)

A：Nomal group B：Model group C：L-Glutamate 25 mg/kg

D：L-Glutamate 50 mg/kg E：L-Glutamate 100 mg/kg F：L-Glutamate 200 mg/kg

G：L-Glutamate 400 mg/kg H：L-Glutamate 800 mg/kg I：Positive control group

Tab.S3 showed the pellets are rod-shaped at spheronization rate of 600 rpm with low sphericity and low yield; when the spheronization rate is 800 rpm, the sphericity of the pellets is higher, but the yield is low; when the spheronization rate is 1000 rpm, the yield and sphericity are both high. Therefore, the spheronization rate of 1000 rpm is selected.

Tab.S3 The effect of spheronization rate on yield and sphericity of aspirin drug-loaded pellets

| Spheronization rate (rpm) | Yield (%) | Sphericity |
| --- | --- | --- |
| 1000 | 86.52 | 0.90 ± 0.04 |
| 800 | 78.93 | 0.92 ± 0.03 |
| 600 | 73.26 | 0.76 ± 0.12 |

In Tab.S1 and Fig.S2, with the extension of the spheronization time, the yield and sphericity of the pellets increase, but the dissolution rate slows down. When the spheronization time is 6 min, the dissolution rate of the pellets is less than 80%, which does not meet the quality requirements. Considering the yield and dissolution of pellets, the spheronization time of 4 min is selected.

Tab.S4 The effect of the time of spheronization on yield and sphericity of aspirin drug-loaded pellets

| Spheronization time (min) | Yield (%) | Sphericity |
| --- | --- | --- |
| 2 | 70.62 | 0.86 ± 0.08 |
| 4 | 82.83 | 0.91 ± 0.02 |
| 6 | 85.21 | 0.93 ± 0.08 |

Fig.S2 The dissolution curve of aspirin drug-loaded pellets with different time of spheronization

Fig.S3 showed that when the weight gain of aspirin enteric-coated pellets was 10%, 15%, and 20%, the release rate in pH 6.8 PBS for 45 min were all greater than 80%, and there were no significant difference. Thus, the weight gain of 10% is selected.

Fig.S3 The effect of the weight gain of enteric layer on release behavior of aspirin enteric-coated pellets in pH 6.8 PBS after placing for 2 h in 0.1 mol/L HCl solution

Tab. S5 showed the compound enteric-coated granules have good acid resistance and meet quality requirements

Tab. S5 Release behavior of aspirin from compound enteric-coated granules

in 0.1 M HCl solution for 2 h

| Batch number | Acid resistance (%) |
| --- | --- |
| 1 | 2.47 ± 0.09 |
| 2 | 2.36 ± 0.11 |
| 3 | 2.56 ± 0.06 |

Fig. S4 showed the cumulative release of aspirin from compound enteric-coated granules in pH 6.8 PBS were greater than 80%, which met the quality requirements.

Fig. S4 Release behavior of aspirin from compound enteric-coated granules

in pH 6.8 PBS after 2 h in 0.1 M HCl solution

Fig. S5 showed the cumulative release of L-glutamate from compound enteric-coated granules in pH 7.2 PBS were greater than 90% met the quality requirements.

Fig. S5 Release behavior of L-glutamate from compound enteric-coated granules

in pH 7.2 PBS
